# A rare case of hemophagocytic lymphohistiocytosis mimicking flare of systemic lupus erythematosus

**DOI:** 10.1002/rcr2.1140

**Published:** 2023-04-11

**Authors:** Areeb Tiwana, Maida Tiwana, Jiang Wang, Muhammad Umair Khawar

**Affiliations:** ^1^ Department of Internal Medicine Nishtar Hospital Multan Pakistan; ^2^ Department of Environmental and Public Health University of Cincinnati Cincinnati Ohio USA; ^3^ Department of Pathology & Lab Medicine University of Cincinnati College of Medicine Cincinnati Ohio USA; ^4^ Department of Internal Medicine, Division of Pulmonary, Critical Care and Sleep Medicine University of Cincinnati Cincinnati Ohio USA

**Keywords:** autoimmune disease, disseminated histoplasmosis, HLH, SLE flare

## Abstract

The Ohio and Mississippi River Valleys are endemic to histoplasmosis. It is usually self‐limiting in immunocompetent people, but it can cause morbidity and mortality if not detected early in people with an underlying autoimmune disease. Disseminated Histoplasmosis induced hemophagocytic lymphohistiocytosis (HLH) mimicking the flare of an underlying autoimmune disease, is uncommon in the published literature. Disseminated histoplasmosis (DH) can cause multiorgan involvement, especially in a patient with an underlying autoimmune disease. We present the case of a 24‐year‐old female with HLH who was initially treated as a flare of autoimmune disease but later etiology was confirmed as disseminated histoplasmosis on bone marrow histopathological examination.

## Graphical abstract

Disseminated histoplasmosis can cause multiorgan involvement especially in a patient with underlying autoimmune disease as in this case of a 24‐year‐old female with HLH who was initially treated as a flare of autoimmune disease but later etiology was confirmed as disseminated histoplasmosis on bone marrow histopathological examination.

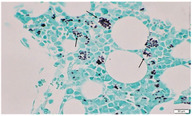

## INTRODUCTION

Hemophagocytic lymphohistiocytosis (HLH) is a rare inflammatory disorder that is often fatal and characterized by the uncontrolled proliferation of lymphocytes and macrophages. It is recognized as one of the cytokine storms, divided into primary and secondary subtypes. Primary HLH is due to genetic defects and secondary is due to infections, malignancies, and autoimmune conditions. We report a unique case of HLH in a 24‐year‐old female with SLE leading to multiorgan failure, that appeared to be related to systemic lupus erythematosus (SLE) flare on presentation, later found to have DH on further evaluation.

## CASE REPORT

A 24‐year‐old female with SLE, not on immunosuppressants, presented to an outside hospital for evaluation of fever, malaise, cough and arthralgia. Initial physical examination was unremarkable.

Laboratory workup was concerning for pancytopenia, low complement activity, and proteinuria. Computed tomography (CT) of the chest revealed bilateral pleural effusions and ground glass opacities, raising concern of an infectious process while an underlying autoimmune disease flare was also on the differential. Her infectious workup remained negative including sputum and blood cultures and she was treated with pulse dose steroids (1 gm methylprednisolone daily for 3 days) for concerns of a flare of her underlying SLE. She was discharged home on azathioprine and hydroxychloroquine but was readmitted to the same hospital after 2 days due to worsening shortness of breath, fatigue and hypotension. Laboratory workup at that time showed worsening pancytopenia with elevated lactate dehydrogenase (LDH), normal haptoglobin, low fibrinogen and coagulopathy. For management of presumed refractory flare of underlying SLE, she was treated again with pulse dose steroids (1 gm methylprednisolone daily for 3 days) and IVIG. During this hospital admission, a bone marrow biopsy was also performed for further evaluation.

**FIGURE 1:**
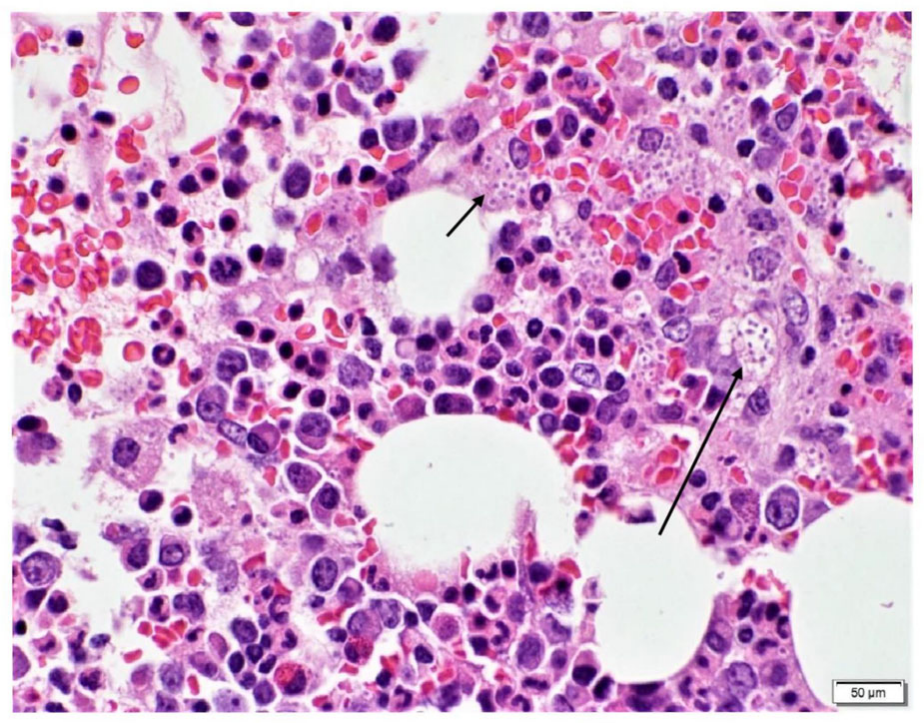
Bone marrow biopsy: H&E stain, showing clusters of fungal yeasts (arrows)

**FIGURE 2:**
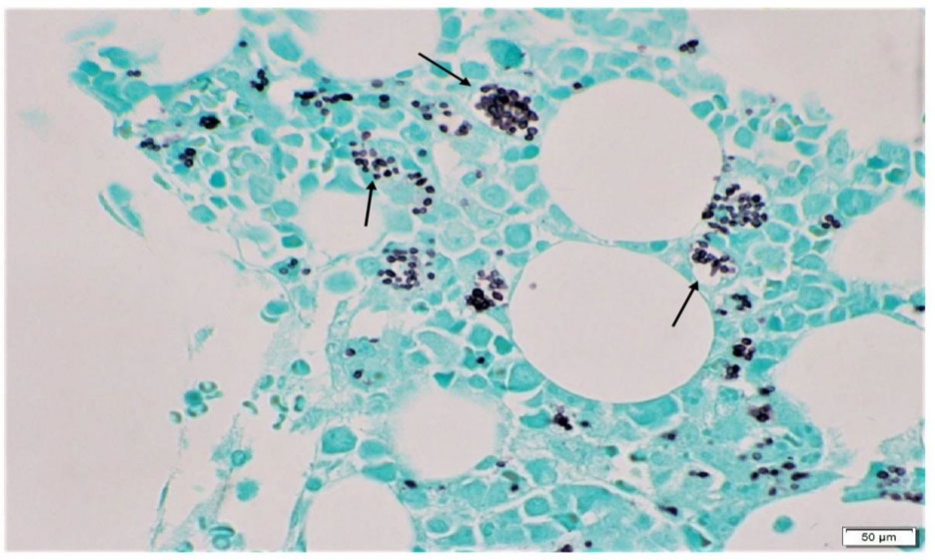
Bone marrow biopsy: GMS stain, showing numerous yeasts of histoplasma capsulatum (arrows)

**FIGURE 3:**
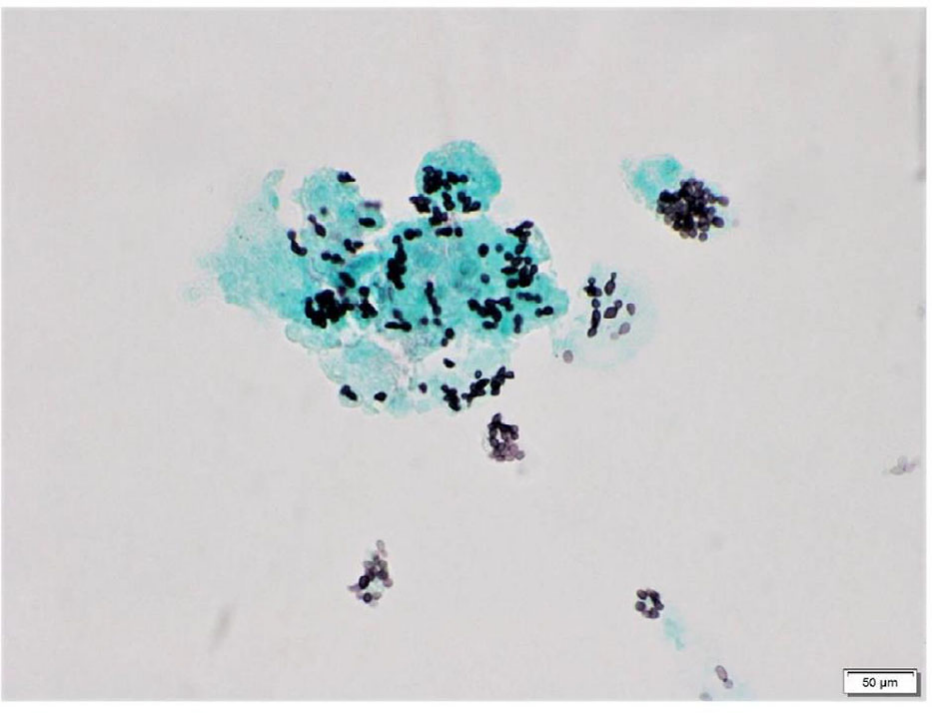
Lung BAL cytology: GMS stain, showing numerous yeasts of histoplasma capsulatum

She was later transferred to our hospital for further evaluation due to lack of clinical improvement. Laboratory work‐up here revealed pancytopenia, high ferritin levels (>7500 ng/mL), reduced complement activity, elevated triglycerides, and decreased fibrinogen level along with fever and hepatomegaly on physical examination. This was concerning for HLH due to underlying autoimmune disease as the initial infectious work‐up has remained negative to date. Her condition deteriorated requiring ICU transfer due to worsening hypoxemia and new onset hemoptysis, requiring urgent endotracheal intubation and mechanical ventilation. A bedside fiberoptic bronchoscopy was performed for evaluation of hemoptysis and serial bronchoalveolar lavage (BAL) aliquots revealed diffuse alveolar haemorrhage (DAH). On discussion with Rheumatology, urgent plasma exchange (PLEX) was planned. However, her bone marrow biopsy from the outside hospital revealed the evidence of disseminated histoplasmosis (Figures 1 and 2). In addition, BAL cytology revealed numerous yeasts of histoplasma capsulatum (Figure 3) and histoplasma antigen was also detected in urine cytology. She was immediately started on liposomal amphotericin therapy and further immunosuppressive treatment including methylprednisolone was held.

The patient was diagnosed with disseminated histoplasmosis leading to HLH. Her condition started improving gradually with aggressive antifungal therapy and after few days, she was successfully extubated and remained off vasopressor support. She was discharged home in stable condition with close Infectious Disease follow‐up.

## DISCUSSION

HLH is a pathologic hyperactive inflammatory response to an ineffective immune system. It is characterized by the overactivity of histiocytes, T cells, natural killer cells and B cells causing production of cytokines leading to cytopenia. HLH secondary to histoplasma can be caused by excessive antigen presentation or cytokine release in an immunocompromised setting. Defence against histoplasma is provided by macrophages and helper t cells that lead to release of cytokines including interferon gamma and TNF alpha to form granulomas. HLH can mimic sepsis, autoimmune lymphoproliferative syndrome or multiorgan failure so prompt recognition and treatment is, therefore, necessary to improve outcomes.

Histoplasmosis is one of the most prevalent mycoses in the Midwestern and Central United States particularly areas around the Ohio and Mississippi River Valleys. Disseminated Histoplasmosis (DH) has been rarely reported in immunocompetent individuals.^1^ It most commonly occurs in the immunocompromised, advancing to multiorgan involvement with pulmonary involvement being the most fatal. Pulmonary histoplasmosis can present as acute or chronic cavitary histoplasmosis, pleural effusion, pericarditis, granulomatous mediastinitis, pneumonia and bronchiectasis. Its detection depends on the extent and exposure of the disease. Histopathology and cytology of specimens from lung or mediastinal lymph node biopsy allow rapid diagnosis of histoplasmosis. Histoplasma antigen detection enzyme immunoassay (EIA) is detected in 40% of cases of acute diffuse pulmonary involvement. Other diagnostic methods include fungal culture of BAL fluid and antibody testing of serum by immunodiffusion and complement fixation.^2^

The diagnostic criteria for HLH in adults includes five of the following nine findings: fever, splenomegaly, bi‐cytopenia, hypertriglyceridemia, hemophagocytosis in bone marrow studies, low or absent natural killer cell activity, elevated ferritin, soluble CD25 or CXCL9 levels.^3^

HLH causing DAH due to disseminated histoplasmosis in patients with SLE has been reported very rarely, with only two cases found in published literature.^4^

Our patient was started on amphotericin B which led to a rapid improvement in her condition. Current recommendations include completing antifungal treatment for at least a year.^3^

This case was challenging as the initial aetiology of HLH was thought to be related to the flare of SLE until bone marrow results revealed presence of histoplasmosis. Therefore, we emphasize that clinicians should always maintain a high index of suspicion of endemic infectious etiologies especially in patients with underlying autoimmune disease, as missing this differential would be catastrophic in the presence of underlying autoimmune disease process.

## AUTHOR CONTRIBUTIONS

All the authors mentioned, contributed equally.

## CONFLICT OF INTEREST STATEMENT

None declared.

## ETHICS STATEMENT

The authors declare that appropriate written informed consent was obtained for the publication of this manuscript.

## Data Availability

The data that support the findings of this study are available from the corresponding author upon reasonable request.
